# miR-29b-3p inhibits post-infarct cardiac fibrosis by targeting FOS

**DOI:** 10.1042/BSR20201227

**Published:** 2020-09-02

**Authors:** Yongliang Xue, Xuefang Fan, Ruobing Yang, Yuanyuan Jiao, Yang Li

**Affiliations:** 1 Dongjing Clinical College of Henan University, Henan Province, China; 2 Department of Cardiology, Xianyang Hospital of Yan’an University, Xianyang, Shaanxi Province, China

**Keywords:** cardiac fibrosis, differentiation, FOS, migration, miR-29b-3p, proliferation

## Abstract

Background: Cardiac fibrosis after myocardial infarction (MI) is a major cause of heart deterioration. Recently, the roles of microRNAs (miRNAs) in various cardiovascular diseases associated with cardiac fibrosis have been extensively investigated. The present study aimed to investigate the role and mechanism of miR-29b-3p in cardiac fibrosis after MI.

Methods: miR-29b-3p expression in TGF-β1-activated cardiac fibroblasts (CFs) was detected by qRT-PCR. Cell Counting Kit-8 (CCK-8) and Trans-well assays were performed to evaluate CFs proliferation and migration ability, respectively. Protein expressions of α-SMA, collagen I, collagen III, MMP2, and MMP9 were examined by Western blot assay. Bioinformatics, luciferase, and RNA immunoprecipitation (RIP) assays were carried out to determine whether FOS was targeted by miR-29b-3p.

Results: TGF-β1 treatment dose-dependently curbed miR-29b-3p expression in CFs. miR-29b-3p restrained the promotive impacts of TGF-β1 on CFs proliferation, migration, and differentiation. FOS was affirmed to be a target of miR-29b-3p, elevated expression of FOS reversed the inhibitory effects of miR-29b-3p on cell proliferation, migration, and differentiation in TGF-β1-activated CFs.

Conclusion: miR-29b-3p degraded the pro-fibrosis effect induced by TGF-β1 via targeting FOS, providing a prospective therapeutic avenue for cardiac fibrosis after MI.

## Introduction

Myocardial infarction (MI) remains a major cause of cardiovascular disease (CVD)-associated death worldwide. The arrest of blood flow to the left ventricular free wall after MI should be responsible for cardiomyocyte loss, ventricle pathological remodeling, and myocardial dysfunction, inevitably leading to post-infarction heart failure and cardiac fibrosis [1]. It is generally accepted that the extracellular matrix (ECM) functions as a major player involving in the communication between cells [2]. In the healthy myocardium, a perfect equilibrium exists between the deposition and degradation of ECM components, which are mediated by a family of matrix metalloproteinases (MMPs). During heart failure, ECM proteins (e.g. collagen I and collagen III) are excessively deposited in response to numerous pathological stimuli, leading to the maladaptive ventricular remodeling in the heart [3,4].

Recently, growing number of evidences have pointed to the implication of transforming growth factor-β1 (TGF-β1) in different forms of physiological and pathological processes, including cell proliferation, migration, differentiation, and apoptosis [5]. Additionally, TGF-β1 is affirmed to be a crucial mediator of cardiac fibrosis via triggering the synthesis of collagen in ECM, as well as the enrichments of MMPs [6,7]. Cardiac fibroblasts (CFs) are a significant and unique population of non-muscle cells in human heart, associated with the development of cardiac fibrosis mainly attributed to its proliferation, migration, and differentiation potential to myofibroblasts during MI [8,9]. Therefore, great efforts devoted to inhibit cardiac fibrosis are important for the prevention and treatment of heart failure after MI.

MicroRNAs (miRNAs) are a type of endogenous small non-coding RNAs that repress gene expression at transcriptional or post-transcriptional level by complementary binding with their 3′ untranslated regions (3′UTRs) [10]. Growing evidence shows that miRNAs play central roles in the development of cardiovascular diseases [11,12]. Moreover, copious miRNAs have been demonstrated to be dysregulated and associated with the progression of cardiac fibrosis. Recently, miR-29b-3p has been attracted a great deal of attentions attributed to its involvement in cardiac pathological processes. A previous study showed that miR-29 contributed to pathological cardiac remodeling and fibrosis by activation of Wnt signaling [13]. Also, miR-29b functioned as a potential therapeutic target for angiotensin II (AngII)-induced cardiac fibrosis by regulation of TGF-β/Smad3 signaling [14].

In view of the implication of miR-29b in the etiology of cardiac fibrosis, we put our attention on the roles and molecular mechanisms of miR-29b-3p in post-MI cardiac fibrosis.

## Methods

### Cardiac fibroblasts (CFs) isolation and culture

Primary CFs used in present study were obtained as previously described [15]. In short, Newborn Sprague-Dawley (SD) rats (2–3 days, male) purchased from Vita River Experimental Animal Technology Company (Beijing, China), After deep anesthesia in the Dongjing Clinical College of Henan University, the rats were killed by cervical dislocation with 2% isoflurane, after which the heart was isolated and ventricles were minced and digested using the mixture of 0.2% collagenase and 0.25% trypsin for 20 min. Digested cells were collected and resuspended in Dulbecco's Modified Eagle Media (DMEM, Gibco, Grand Island, CA, U.S.A.) supplemented with 10% fetal bovine serum (FBS, Gibco). After incubated under a standard condition of 5% CO_2_ at 37°C for 90 min, floating cells were rinsed and attached cells were used as CFs. The cells were grown in DMEM medium containing 10% FBS and 1% penicillin/streptomycin and cultured in a humidified atmosphere with 5% CO_2_ at 37°C. Cells at passage 2-5 were used for the subsequent study. These protocols were performed in line with the Guidelines for Care and Use of Laboratory Animal and approved by the Ethics Committee of Animal Research of Dongjing Clinical College of Henan University.

### Plasmid and cell transfection

miR-29b-3p mimics (miR-29b-3p), miR-29b-3p inhibitor (Anti-miR-29b-3p), small interfering RNA (siRNA) targeting FOS (siFOS), pcDNA-FOS overexpression plasmid (FOS), and relevant negative controls were all obtained from GenePharma Co.,Ltd (Shanghai, China). Oligonucleotides or plasmids were transiently transfected into treated or untreated CFs using Lipofectamine 2000 (Invitrogen, Carlsbad, CA, U.S.A.). Forty-eight hours thereafter, cells were harvested for further analysis.

### qRT-PCR

Total RNA in CFs were extracted using Trizol reagent (Invitrogen, Carlsbad, CA, U.S.A.) referring to the manual provided by manufacturer, followed by the determination of RNA quality and purity using a NanoDrop 2000 spectrophotometer (Thermo Fisher, Waltham, MA, U.S.A.). For miR-29b-3p expression, total RNA was reverse transcribed into cDNA by using TaqMan® miRNA reverse transcription kit (Thermo Fisher), with U6 snRNA as a housekeeping gene. For FOS mRNA expression, M-MLV Reverse Transcriptase Kit (Thermo Fisher) was employed to generate relative cDNA, with β-actin as a housekeeping gene. Afterwards, qRT-PCR was performed using SYBR Green PCR Master Mix (Thermo Fisher) and ran on the Applied Biosystems 7500 Real-time PCR Systems (Applied Biosystems, Foster City, CA, U.S.A.). The expressions of miR-29b-3p and FOS mRNA were calculated using the 2^–ΔΔCt^ method. Special primers were displayed as below. FOS: 5′-ATGATGTTCTCGGGTTTCAA-3′ (forward) and 5′-TGACATGGTCTTCACCACTC-3′ (reverse), β-actin: 5′-CTCTGTGTGGATTGGTGGCT-3′ (forward) and 5′-CGCAGCTCAGTAACAGTCCG-3′ (reverse). Primers for miR-29b-3p and U6 were provided by GenePharma Co.,Ltd.

### Western blot

CFs were lysed for protein extraction by using RIPA buffer (Beyotime, Shanghai, China) mixed with 1% protease inhibitor (Sigma-Aldrich, St. Louis, MO, U.S.A.). Equal amounts of protein from each cell lysate were divided on SDS-PAGE gel and transferred onto polyvinylidene Fluoride (PVDF) membranes (Millipore, Billerica, MA, U.S.A.). After blocked with 5% skim milk for 2 h at 37°C, the membranes were incubated with primary antibodies against α-SMA (1:1000, Cell Signaling Technology, Danvers, MA, U.S.A.), collagen I (1:2000, Abcam, Cambridge, MA, U.S.A.), collagen III (1:5000, Abcam), MMP2 (1:2000, Abcam), MMP9 (1:1000, Abcam), and GAPDH (1:2000, Abcam) overnight at 4°C. Next, the membranes were washed with TBST buffer for three times, and further hatched with HRP-labeled secondary antibody (Abcam) for 1.5 h at 37°C. Protein blots were visualized using enhanced chemiluminescence (ECL, GE Healthcare, Little Chalfont, U.K.). The relative expression levels of proteins were quantified by densitometry with GAPDH as an internal control.

### Cell proliferation

Cells proliferation activity was analyzed by using a Cell Counting Kit-8 (CCK-8, Dojindo, Kumamoto, Japan) according to the manufacturer’s instructions. Briefly, CFs were plated into 96-well plates and cultured in 100 μl medium at a density of 3000 cells/well. Then, 10 µl CCK-8 reagent was added and cells were incubated for another 4 h, followed by the measurement of the absorbance at a wavelength of 450 nm on a Microplate Reader (Thermo Fisher).

### Cell migration

Cell migration assay was carried out using a Trans-well chamber (BD Biosciences, San Jose, CA, U.S.A.). After transfection, resuspended CFs in serum-free medium were seeded on to the upper chamber. Complete medium containing 10% FBS was added to the bottom chamber as a chemoattractant. After 16 h incubation, cells on the upper surface were scrubbed carefully using a swab, and migrated to the lower surface were fixed and strained with 0.1% Crystal Violet (Beyotime). Migratory cells in random five visual fields per chamber were observed and counted under a microscope (Olympus, Tokyo, Japan).

### Luciferase assay

FOS gene was predicted to be a target of miR-29b-3p using Tagerscan online software. For luciferase assay, the partial sequences of FOS 3′UTR containing putative miR-29b-3p binding sites were inserted into psiCHECK^™^-2 luciferase vector (Promega, Madison, WI, U.S.A.) to form the wile-type luciferase reporter (FOS-wt). The mutant luciferase reporter (FOS-mut) containing a point-mutated miR-29-3p binding region was generated using Phusion Site-Directed Mutagenesis Kit (Thermo Fisher). After that, constructed luciferase reporter FOS-wt or FOS-mut was transfected into CFs cells together with miR-NC or miR-29b-3p. Forty-eight hours thereafter, the cells were harvested and luciferase activity was evaluated by Pierce^™^ Renilla-Firefly Luciferase Dual Assay Kit (Thermo Fisher).

### RNA immunoprecipitation (RIP)

Magna RIP RNA-Binding Protein Immunoprecipitation Kit (Millipore, Billerica, MA, U.S.A.) was used to determine the true interaction between FOS and miR-29b-3p. Briefly, CFs transfected with miR-29b-3p or miR-NC were lysed in RIP buffer and the extraction was hatched with ProteinA/G magnetic beads bound with anti-Ago2 (Abcam) or anti-IgG (Abcam). After removal of protein and DNA in immunoprecipitant complex, the enrichment levels of FOS were measured by qRT-PCR.

### Statistical analysis

All data were displayed as the mean ± standard deviation (SD) from three independent experiments. Statistical difference in groups was evaluated by Student’s *t* test or one way ANOVA using SPSS 22.0 software (SPSS, Inc., Chicago, IL, U.S.A.). Statistical significance was considered as *P* values less than 0.05.

## Results

### miR-29b-3p was declined in TGF-β1-stimulated CFs

**Figure 1:**
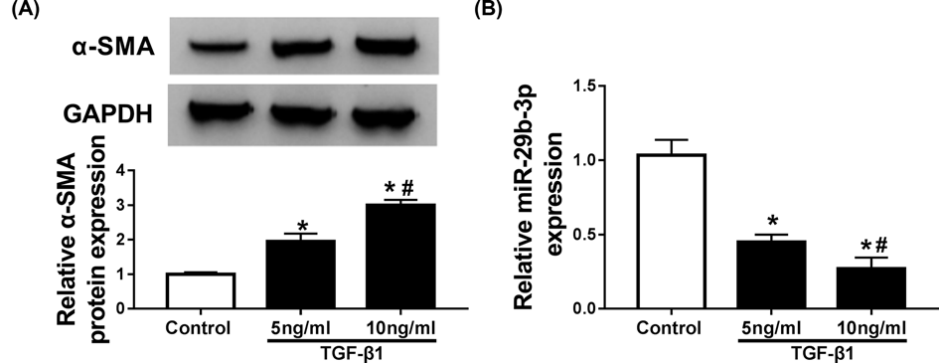
miR-29b-3p was downregulated in TGF-β1-stimulated CFs CFs were stimulated with different concentrations (0, 5, 10 ng/ml) of TGF-β1 for 24 h (**A**) α-SMA protein expression was detected by Western blot assay. (**B**) miR-29b-3p abundance was determined by qRT-PCR. *P<0.05 compared with control, ^#^*P*<0.05 compared with TGF-β1 (5 ng/ml).

TGF-β1 has been individuated as the primary inducer of CFs conversion into myofibroblast. Thus, varying concentrations of TGF-β1 were employed to stimulate the differentiation of CFs. As a result, addition of TGF-β1 contributed to the expression of myofibroblasts marker α-SMA in CFs in a dose-dependent manner (Figure 1A). Moreover, miR-29b-3p kept decreased in TGF-β1-stimulated CFs (Figure 1B). These findings indicated the possible involvement of miR-29b-3p in cardiac fibrosis.

### miR-29b-3p negatively regulated cell proliferation, migration, and myofibroblast differentiation in CFs

**Figure 2:**
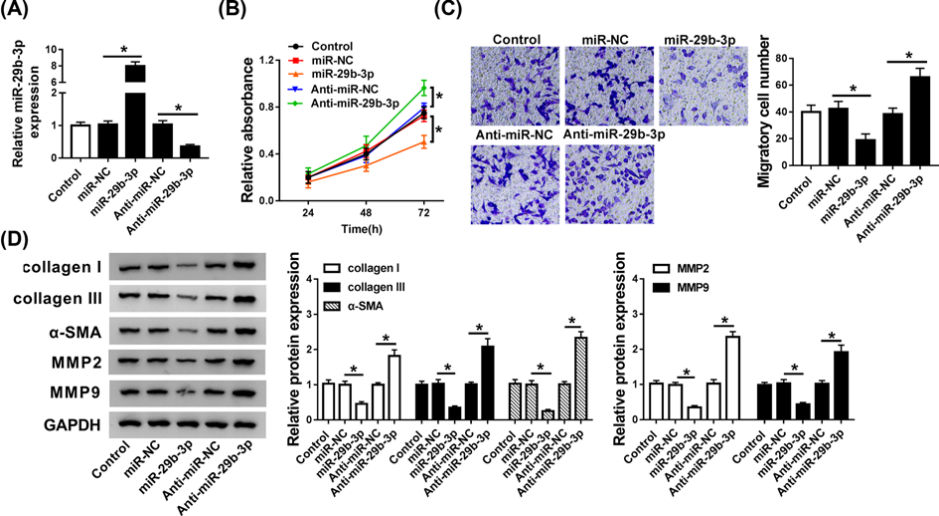
miR-29b-3p expression was negative correlation with CFs proliferation, migration, and differentiation CFs were transfected with miR-NC, miR-29b-3p, Anti-miR-NC, or Anti-miR-29b-3p in CFs. (**A**) The expression level of miR-29b-3p was determined by qRT-PCR. (**B** and **C**) Cell proliferation and migration were determined by CCK-8 and Trans-well assays. (**D**) The abundances of collagen I, collagen III, α-SMA, MMP2, and MMP9 proteins were examined by Western blot assay. *P<0.05 compared with corresponding control.

Enhanced cell proliferation, migration, and differentiation are the major features of CFs activation. To explore the role of miR-29b-3p in CFs activation, we overexpressed or inhibited miR-29-3p through transfection of miR-29-3p mimics or anti-miR-29-3p into CFs (Figure 2A). Cell proliferation and migration detected by CCK-8 or Trans-well showed that transfection of miR-29-3p caused significant proliferation and migration retardation, while miR-223 inhibitor had contrasting effects (Figure 2B,C). Western blot analysis showed that transfection of miR-29b-3p inhibited fibrosis-related proteins collagen I, collagen III, α-SMA expression, while miR-29-3p inhibitor enhanced the expressions of these proteins (Figure 2D). Matrix metalloproteinases (MMPs) are emerged as potential targets in heart dysfunction. MMP-2 and MMP-9, also known as type IV collagenases, contain fibronectin-like domains for collagen binding, and have high expression in cardiac fibrosis [16]. Here, we also observed abated abundances of MMP2 and MMP9 in miR-29b-3p-overexpressed CFs (Figure 2B,C). However, anti-miR-29-3p introduction augmented MMP2 and MMP9 expression (Figure 2D). Taken together, miR-29b-3p restrained CFs proliferation, migration, and differentiation, exerting marked anti-fibrosis effect in cardiac fibrosis.

### miR-29b-3p weakened the pro-fibrosis effect induced by TGF-β1 *in vitro*

**Figure 3:**
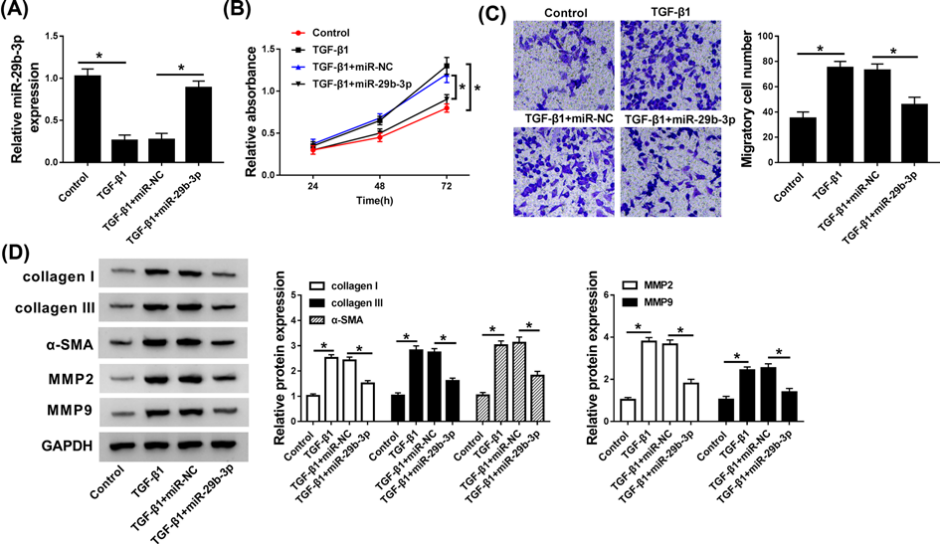
miR-29b-3p weakened the stimulatory effects of TGF-β1 on cell proliferation, migration, and myofibroblast differentiation in CFs CFs treated with TGF-β1 (10 ng/ml) were transfected with miR-NC or miR-29b-3p. (**A**) The abundance of miR-29b-3p was measured by qRT-PCR. (**B** and **C**) CCK-8 and Trans-well assays were performed to assess cell proliferation and migration. (**D**) The protein expressions of collagen I, collagen III, α-SMA, MMP2, and MMP9 were examined by Western blot assay. *P<0.05 compared with corresponding control.

In present study, we attempted to investigate whether miR-29b-3p attenuated the fibrotic process via serving as a downstream effector of TGF-β1 in CFs. CFs cells treated with 10 ng/ml TGF-β1 were transfected with miR-NC or miR-29b-3p, with untreated group with a control. As displayed in Figure 3A, the abundance of miR-29b-3p, detected by qRT-PCR, was decreased in TGF-β1-stimulated CFs, while transfection of miR-29b-3p mimics reversed the inhibitory effect of TGF-β1 on miR-29b-3p expression. Functionally, CCK-8 and Trans-well analyses exposed that TGF-β1 treatment intensified cell proliferation and migration in CFs, but re-expression of miR-29b-3p weakened the effects of TGF-β1 (Figure 3B,C). Moreover, TGF-β1 introduction resulted in the enhancement of collagen I, collagen III, α-SMA, MMP2, and MMP9 protein expression, while pre-transfecting CFs with miR-29b-3p mimics distinctly abrogated the stimulatory effects of TGF-β1 on these proteins expression (Figure 3D). In sum, miR-29b-3p functioned as a negative regulator in TGF-β1-stimulated fibrotic process.

### FOS was a bona fide target of miR-29b-3p

**Figure 4:**
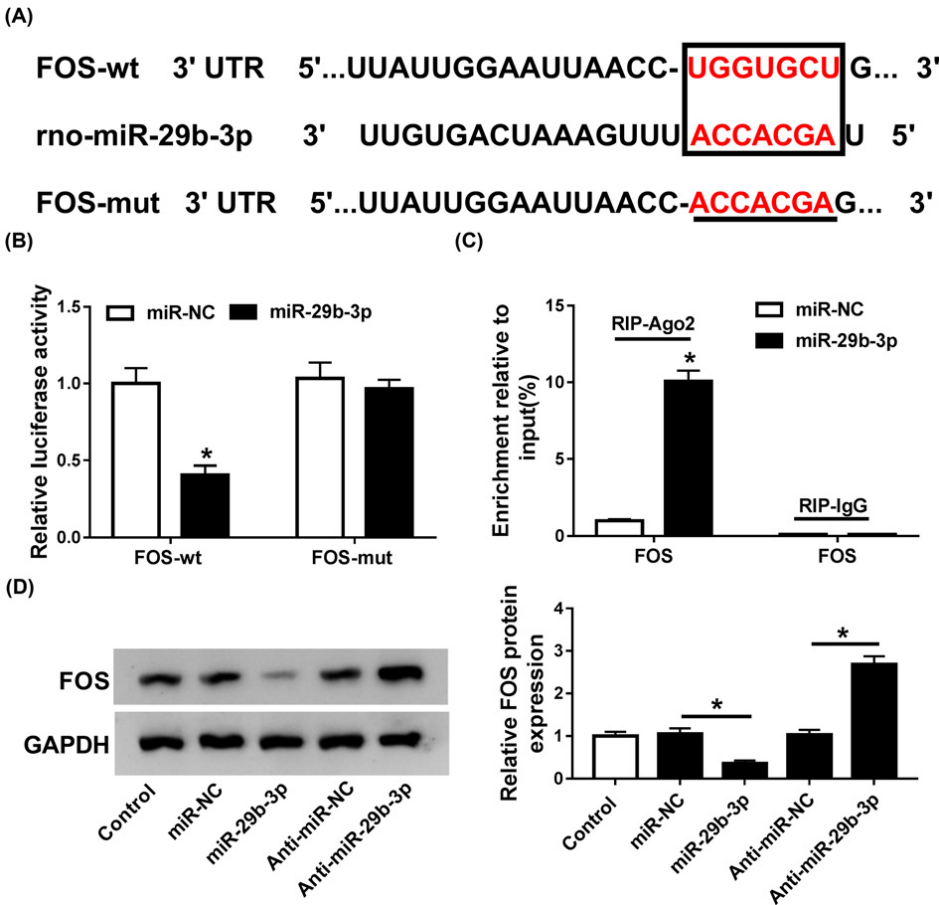
FOS was targeted by miR-29b-3p (**A**) The putative binding regions of miR-29b-3p in the 3′UTR of FOS were predicted using Targetscan software. (**B**) Luciferase activity of FOS-wt or FOS-mut reporter in CFs transfected with miR-NC or miR-29b-3p was evaluated by luciferase assay. (**C**) The correlation between miR-29b-3p and FOS in CFs was detected by RIP assay. (**D**) The protein levels of FOS in miR-29b-3p-overexpressed or inhibited CFs were detected by Western blot assay. *P<0.05 compared with corresponding control.

Targetscan online software was used to identify the candidate target of miR-29b-3p, and results validated the existence of complementary sites between miR-29b-3p and FOS 3′UTR (Figure 4A). Therefore, we further explored whether FOS was targeted by miR-29b-3p. The results of luciferase assay revealed that supplement of miR-29b-3p obviously degraded the luciferase activity of FOS-wt in CFs relative to miR-NC group. However, no significant change was observed on the luciferase activity of FOS-mut (Figure 4B). RIP assay disclosed that FOS was copiously enriched by Ago2 antibody in miR-29b-3p-overexpressed CFs compared to control group, while IgG antibody failed to enrich FOS (Figure 4C). Indeed, FOS protein expression was evidently down-regulated by miR-29b-3p overexpression in CFs compared with miR-NC group, while miR-29b-3p silencing led to opposite augmenting effect on FOS expression (Figure 4D). In a word, these data showed that FOS could be directly targeted by miR-29b-3p in CFs.

### Knockdown of FOS was involved in the fibrosis process mediated by TGF-β1 *in vitro*

**Figure 5:**
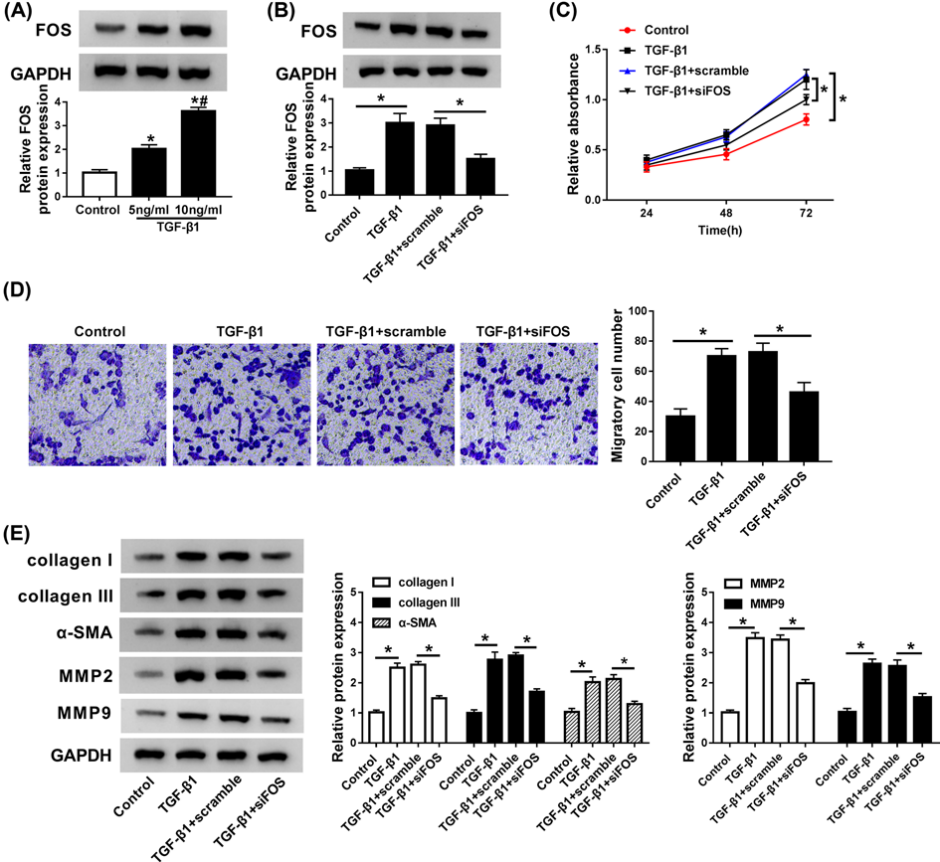
FOS knockdown overturned the stimulatory effects of TGF-β1 on cell proliferation, migration, and myofibroblast differentiation in CFs (**A**) CFs were stimulated with different concentrations (0, 5, 10 ng/ml) of TGF-β1 for 24 h, and FOS protein expression was detected by Western blot assay. (**B**) CFs treated with TGF-β1 (10 ng/ml) were transfected with scramble or si-FOS, followed by the detection of FOS protein expression by qRT-PCR. (**C** and **D**) Cell proliferation and migration ability were assessed by CCK-8 and Trans-well assays. (**E**) The abundances of collagen I, collagen III, α-SMA, MMP2, and MMP9 proteins were measured by Western blot assay. *P<0.05 compared with corresponding control, ^#^*P*<0.05 compared with TGF-β1 (5 ng/ml).

Here, we performed a series of experiments to clarify whether FOS participated in TGF-β1-mediated fibrosis process. The expression of FOS protein kept increased in TGF-β1-stimulated CFs in a dose-dependent way (Figure 5A). Moreover, transfection of si-FOS overturned the stimulatory effect of TGF-β1 on FOS protein expression in CFs (Figure 5B), indicating that si-FOS could be used for the following loss-of-function experiments. CCK-8 and Trans-well analyses showed that depletion of FOS suppressed cell proliferation and migration stimulated by TGF-β1 in CFs (Figure 5C,D). Additionally, the enrichments of collagen I, collagen III, α-SMA, MMP2, and MMP9 proteins, initially induced by TGF-β1, were markedly down-regulated following si-FOS treatment in CFs (Figure 5E). Together, FOS might play a regulatory role in the pro-fibrotic process of TGF-β1.

### miR-29b-3p mediated TGF-β1-stimulated fibrosis process via targeting FOS

**Figure 6:**
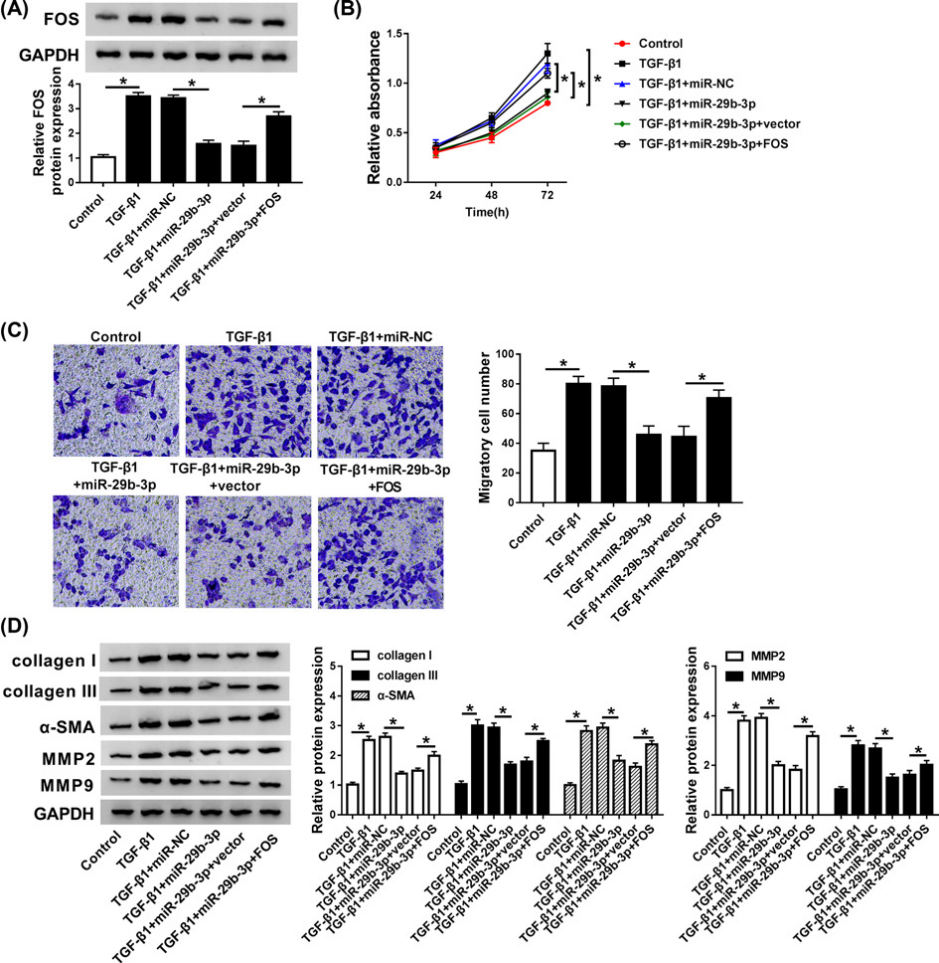
miR-29b-3p mediated cell proliferation, migration, and myofibroblast differentiation in TGF-β1-activated CFs via targeting FOS CFs treated with TGF-β1 (10 ng/ml) were transfected with miR-NC, miR-29b-3p, miR-29b-3p+vector, or miR-29b-3p+FOS. (**A**) The expression of miR-29b-3p was examined by qRT-PCR. (**B** and **C**) CCK-8 and Trans-well analysis was performed to evaluate cell proliferation and migration ability. (**D**) The enrichments of collagen I, collagen III, α-SMA, MMP2, and MMP9 proteins were measured by Western blot assay. *P<0.05 compared with corresponding control.

To further elucidate the mechanism underlying how miR-29b-3p mediated fibrotic process, rescue experiments were carried out by transfection of miR-NC, miR-29b-3p, miR-29b-3p+vector, or miR-29b-3p+FOS into TGF-β1-stimulated CFs. Obviously, miR-29b-3p addition suppressed the expression FOS protein in TGF-β1-activated CFs, while transfection of FOS-overexpressed plasmid abrogated the inhibitory effect of miR-29b-3p on FOS expression (Figure 6A), implying high transfection efficiency in each case. Moreover, FOS enrichment enhanced cell proliferation and migration initially suppressed by miR-29b-3p in TGF-β1-treated CFs, as demonstrated by CCK8 and Trans-well assays (Figure 6B,C). Furthermore, Western blot analysis revealed that restoration of FOS reversed the inhibitory effects of miR-29b-3p on collagen I, collagen III, α-SMA, MMP2, and MMP9 protein expression in TGF-β1-stimulated CFs (Figure 6D). These data indicated that miR-29b-3p was involved in TGF-β1-mediated fibrosis process via repressing FOS.

## Discussion

Cardiac fibrosis after MI is a significant pathological feature in heart failure, and contributes to the distorted heart architecture and function [17]. Abnormal activation and proliferation of CFs should be responsible for the development of Cardiac fibrosis [18]. Our study observed a notable down-regulation of miR-29b-3p and up-regulation of FOS in TGF-β1-induced cardiac fibrosis. Elevated expression of miR-29b-3p impeded the process of cardiac fibrosis induced by TGF-β1 through blockade of CFs proliferation, migration, and differentiation toward to myofibroblast, indicating the anti-fibrosis effect of miR-29b-3p. Our data also strongly validated that miR-29b-3p functioned as a direct epigenetic regulator of FOS and negatively regulated FOS expression in CFs.

Accumulating evidences have confirmed the critical regulatory roles of miRNA in the progression of cardiac fibrosis. For instance, forced expression of miR-433 contributed to cardiac fibrosis and ventricular dysfunction following myocardial infarction possibly through targeting AZIN1 and JNK1, which functioned as an anti-fibrosis factor in the progression of cardiac fibrosis [19]. Also, miR-155 deficiency attenuated cardiac fibrosis triggered by diabetes via regulation of TGF-β1-Smad2 signaling pathway [20]. In addition, up-regulation of miR-21 facilitated the impacts of TGF-β1-induced CFs activation and cardiac fibrosis after MI through targeting TGF-β/Smad7 signaling pathway [21]. MiR-29b is a widely-known miRNA associated with the development of tissue fibrosis. As reported by Roderburg et al., miR-29b showed significant low expression in patients with advanced liver cirrhosis, exogenous restoration of miR-29b alleviated the liver fibrosis via down-regulating collagen in murine hepatic stellate cells (HSC) [22]. Wei et al. [23] disclosed that TGF-β/Smads signaling stimulated the development of renal fibrosis by suppressing miR-29. H19 exhibited promotive effect on bleomycin (BLM)-induced idiopathic pulmonary fibrosis (IPF) via interacting with miR-29b [24]. In present study, we observed a low expression of miR-29b-3p in TGF-β1-activated CFs. Functionally, overexpression of miR-29b-3p prevented the promotive effects of TGF-β1 on CFs proliferation, migration, and CFs collagen production, leading to the remission of cardiac fibrosis. Consistent with our findings, the inhibitory effects of miR-29b-3p on cardiac fibrosis also be validated by Zhang et al. [14] and Panizo et al. [25].

As mentioned above, a broad range of miRNAs are linked with the development of cardiac fibrosis mainly through targeting the fibrosis-associated genes. c-FOS, encoded by FOS gene, dimerizes with c-Jun to form activator protein-1 (AP-1) [26]. Importantly, FOS been reported to participate in various pathological processes, including brain injury [27,28], angiopoiesis [29], and malignancies [30,31]. Also, c-Fos was confirmed to act as a major regulator in tissue fibrosis. Up-regulation of c-Fos by TGF-α induced the transcription of TGF-β1, a key pro-fibrosis cytokine [32]. Acetaldehyde triggers procollagen I and fibronectin gene transcription in rat fatstoring cells (FSC) possibly by up-regulation of c-Fos and c-Jun, thus leading to the aggravation of hepatic fibrosis [33]. Moreover, globular adiponectin activates AP-1 pathway via enhancing c-Fos and c-Jun expression, which eventually resulted in the development of cardiac fibrosis [34]. Forced expression of miR-101a/b suppressed the proliferation and collagen production of AngII-induced rat CFs via inactivation of c-Fos/TGF-β1 pathway [35]. Despite the regulation of c-Fos in cardiac fibrosis, there remains no direct evidence to confirm whether miR-29b-3p is involved in the pathogenesis of post-MI heart failure via targeting FOS. Here, we provided the first evidence that FOS was an authentic target of miR-29b-3p. Similar to miR-29b-3p overexpression, knockdown of FOS inhibited the proliferation, migration, and differentiation of TGF-β1-activated CFs. Inversely, restoration of FOS weaked the inhibitory effects of miR-29b-3p on TGF-β1-induced CFs proliferation, migration, and differentiation.

Taken together, our study elucidated the anti-fibrotic impact of miR-29b-3p on cardiac fibrosis partly by targeting FOS. These findings indicated that exogenous application of miR-29b-3p might be a promising intervention in the management of post-MI heart failure.

## Data Availability

Please contact the correspondence author for the data request.

## Abbreviations

AngII: angiotensin II
AP-1: activator protein-1
CCK-8: Cell counting Kit-8
CF: cardiac fibroblast
CVD: cardiovascular disease
ECM: extracellular matrix
FSC: fatstoring cells
HSC: hepatic stellate cells
MI: myocardial infarction
MMP: matrix metalloproteinase
PVDF: polyvinylidene fluoride
RIP: RNA immunoprecipitation
SD: Sprague-Dawley
siRNA: small interfering RNA
TGF-β1: transforming growth factor -β1
